# Procedural analgesic interventions in China: a national survey of 2198 hospitals

**DOI:** 10.1186/s12871-022-01783-6

**Published:** 2022-08-06

**Authors:** Yafeng Wang, Feng Xu, Shuai Zhao, Linlin Han, Shiqian Huang, Hongyu Zhu, Yuanyuan Ding, Lulin Ma, Wenjing Zhao, Tianhao Zhang, Xiangdong Chen, Pain Group of Chinese Society of Anesthesiology, Yi Feng, Tieli Dong, Zhonghuang Xu, Yan Lv, Zhen Hua, Yanhong Liu, Yanyan Bai, Song Cao, Yajun Chen, Jianhua Du, Yinghui Fan, Guang Han, Nong He, Xingying He, Yongjin He, Yanhui Hu, Yanhua Li, Dezhao Liu, Ping Liu, Silan Liu, Danxu Ma, Minyu Ma, Fei Ren, You Shang, Xiaofeng Shen, Jie Song, Muer Tie, Chunhui Wang, Feng Wang, Haitang Wang, Huishu Wang, Tiancheng Wang, Yaping Wang, Wei Wu, Hua Xu, Zhaoxia Xue, Lingzhi Yu, Leyun Zhan, Dong Zhang, Jinjun Zhang, Duozhi Wu, Dong Yang

**Affiliations:** 1 Department of Anesthesiology, Union Hospital, Tongji Medical College, Huazhong University of Science and Technology, Wuhan, 430022 China; 2 Peking University People’s Hospital, Beijing, China; 3 The Second Affiliated Hospital of Zhengzhou University, Zhengzhou, China; 4 Peking Union Medical College Hospital, Chinese Academy of Medical Sciences and Peking Union Medical College, Beijing, China; 5 Xijing Hospital, Fourth Military Medical University, Shaanxi Xi’an, China; 6 Beijing Hospital, Chinese Academy of Medical Sciences, Beijing, China; 7 The First Medical Center of Chinese PLA General Hospital, Beijing, China; 8 The First Hospital of Hohhot, Hohhot, China; 9 Affiliated Hospital of Zunyi Medical University, Zunyi, China; 10 Tianjin Medical University General Hospital, Tianjin, 300052 China; 11 The Second Affiliated Hospital to Xinjiang Medical University, Urumqi, China; 12 Renji Hospital, School of Medicine, Shanghai Jiaotong University, Shanghai, China; 13 Shengjing Hospital of China Medical University, Shenyang, China; 14 Beijing Friendship Hospital, Capital Medical University, Beijing, China; 15 Second Affiliated Hospital of Naval Medical University, Shanghai, China; 16 Tianjin First Central Hospital, Tianjin, China; 17 Second Affiliated Hospital of Nanchang University, Nanchang, China; 18 The First People’s Hospital of Yunnan Province, Kunming, China; 19 The third affiliated hospital of Sun Yat-sen university, Guangzhou, China; 20 First Affiliated Hospital of Soochow University, Suzhou, China; 21 Beijing Chaoyang Hospital, Capital Medical University, Beijing, China; 22 The First Affiliated Hospital of Zhengzhou University, Zhengzhou, China; 23 Xiangya Hospital, Central South University, Changsha, China; 24 The First Affiliated Hospital of Jinzhou Medical University, Jinzhou, China; 25 Woman’s Hospital of Nanjing Medical University, Nanjing, China; 26 The Second Affiliated Hospital of Nantong University, Nantong, Jiangsu China; 27 Inner Mongolia Autonomous Region People’s Hospital, Hohhot, China; 28 The Forth Affiliated Hospital of Anhui Medical University, Hefei, China; 29 General Hospital of Ningxia Medical University, Yinchuan, China; 30 Nanfang Hospital of Southern Medical University, Guangzhou, China; 31 Union Hospital, Fujian Medical University, Fuzhou, China; 32 The People’s Hospital of Liaoning Province, Shenyang, China; 33 The Second Xiangya Hospital of Central South University, Changsha, China; 34 The General Hospital of Western Theater Command, Chengdu, China; 35 Changhai Hospital, The Second Military Medical University, Shanghai, China; 36 The First Hospital of Shanxi Medical University, Taiyuan, China; 37 Jinan Central Hospital, Shandong University, Jinan, China; 38 The First People’s Hospital of Yichang, Yichang, China; 39 Hebei General Hospital, Shijiazhuang, China; 40 The First Affiliated Hospital of Sun Yat-sen University, Guangzhou, China; 41 Hainan General Hospital, Haikou, China

**Keywords:** Sedation, Analgesia, Survey, China, Procedural analgesic interventions

## Abstract

**Background:**

Humane treatment requires the provision of appropriate sedation and analgesia during medical diagnosis and treatment. However, limited information is available about the status of procedural analgesic interventions in Chinese hospitals. Therefore, a nationwide survey was established to identify challenges and propose potential improvement strategies.

**Methods:**

Forty-three members of the Pain Group of Chinese Society of Anesthesiology established and reviewed the questionnaire, which included (1) general information on the hospitals, (2) the sedation/analgesia rate in gastrointestinal endoscopy, labor, flexible bronchoscopy, hysteroscopy in China, (3) staff assignments, (4) drug use for procedural analgesic interventions, and (5) difficulties in procedural analgesic interventions. The data were obtained using an online questionnaire sent to the chief anesthesiologists of Chinese hospitals above Grade II or members of the Pain Group of Chinese Society of Anesthesiology.

**Results:**

Valid and complete questionnaires were received from 2198 (44.0%) hospitals, of which 64.5% were Grade III. The overall sedation/analgesia rates were as follows: gastroscopy (50.6%), colonoscopy (53.7%), ERCP (65.9%), induced abortion (67.5%), labor (42.3%), hysteroscopy (67.0%) and fiber bronchoscopy (52.6%). Compared with Grade II hospitals, Grade III hospitals had a higher proportion of procedural analgesic interventions services except for induced abortion. On average (median [IQR]), each anesthesiologist performed 5.7 [2.3—11.4] cases per day, with 7.3 [3.2—13.6] performed in Grade III hospitals and 3.4 [1.8—6.8] performed in Grade II hospitals (z = -7.065, *p* < 0.001).

**Conclusions:**

Chinese anesthesiologists have made great efforts to achieve procedural analgesic interventions, as evidenced by the increased rate. The uneven health care provided by hospitals at different levels and in different regions and the lack of anesthesiologists are the main barriers to optimal procedural analgesic interventions.

**Supplementary Information:**

The online version contains supplementary material available at 10.1186/s12871-022-01783-6.

## Introduction

A remarkable achievement in the economic and health care systems of China has been made over the past few decades in which health care services have transformed from basic medical care into high-quality and comfortable medical care, which is based on a foundation of procedural analgesic interventions [1]. The American Society of Anesthesiologists stated that during labor, maternal request is a sufficient medical indication for pain relief in the absence of a medical contraindication, and previous studies have shown that sedation/analgesia applied during colonoscopy leads to better results [2, 3]. A survey was performed on the use of neuraxial analgesia for pain relief during labor and sedation for pain relief during gastrointestinal endoscopy in the United States, and the rates were 73% and 74%, respectively. [4, 5]. The sedation rates for gastrointestinal endoscopy were reported to be 78% for colonoscopy and 100% for endoscopic retrograde cholangiopancreatography (ERCP) in Greece (2009) [6] and 82% for gastroscopies and 91% for colonoscopies in Germany (2013) [7].

Importantly, the anesthesiologist is essential for achieving procedural analgesic interventions. The National Health Commission of the People’s Republic of China has focused on anesthesia and analgesia outside the operating room [8]. However, to date, the current status of procedural analgesic interventions of gastrointestinal endoscopy, labor, induced abortion, flexible bronchoscopy and hysteroscopy in China is poorly understood. Herein, we conducted a national survey to investigate the status of procedural analgesic interventions in China, identify the challenges, and propose potential improvement strategies.

## Methods

### Population

Four thousand nine hundred ninety-six hospitals above Grade II from 31 provinces and municipalities across mainland China, which was representative of the situation in Chinese hospitals, were identified from the National Health Commission of the People’s Republic of China as we described previously [9]. Grade II hospitals are defined as centers that provide medical and health services across several communities and represent regional technical centers, while Grade III hospitals are defined as medical prevention technology centers with comprehensive medical, teaching and scientific research capacities [1, 9].

### Questionnaire design and conduct of the survey

Our questionnaire was established by 43 members of the Pain Group of the Chinese Society of Anesthesiology after referring to surveys from England, the United States and China [1, 10–12]. All these members were from Grade III hospitals and experts in pain management, most of them were chief anesthesiologists or associate chief anesthesiologists. The questionnaire included (1) general information for the hospitals, (2) sedation/analgesia rate used for gastrointestinal endoscopy, labor, flexible bronchoscopy, and hysteroscopy in China, (3) staff assignments, and (4) drug use for procedural analgesic interventions. Additionally, we collected information about the difficulties associated with procedural analgesic interventions. The questionnaire was subsequently distributed to the chief anesthesiologist or a member of the Pain Group of the Chinese Society of Anesthesiology in each identified hospital through WeChat (Tencent, Shenzhen, China) as we described previously [9]. In case of no response, second or third calls were performed. WeChat software is a free application that provides instant messaging services for smart terminals, and it has more than 1.08 billion active users per month. Data collection was completed from March 1st to November 1st in 2019.

Because our questionnaire was a descriptive survey and the answers were mainly obtained from annual/monthly quality reports by each department of anesthesiology, we did not perform reliability and validity tests as recommended by Story et al. [13]. Questionnaires were excluded if the response times were less than 10 min. In addition, this national survey mainly focused on the quality control of the department of anesthesiology in each surveyed hospital and personally identifiable information or clinical outcome was not collected; hence, this study was not considered a clinical trial, and ethics committee approval was not needed.

### Statistical analysis

Once the questionnaire was submitted, data was automatically uploaded to Microsoft Office Excel (Microsoft, USA) and checked for errors. Data collection was completed by November 2019. Statistical analyses were performed by the SPSS 24.0 software (IBM, USA). The chi-squared test or Mann–Whitney U test was utilized to assess differences between Grade III and Grade II hospitals based on data types and *P* < 0.05 was considered statistically significant in this study.

## Results

### Characteristics of the surveyed hospitals

**Fig. 1:**
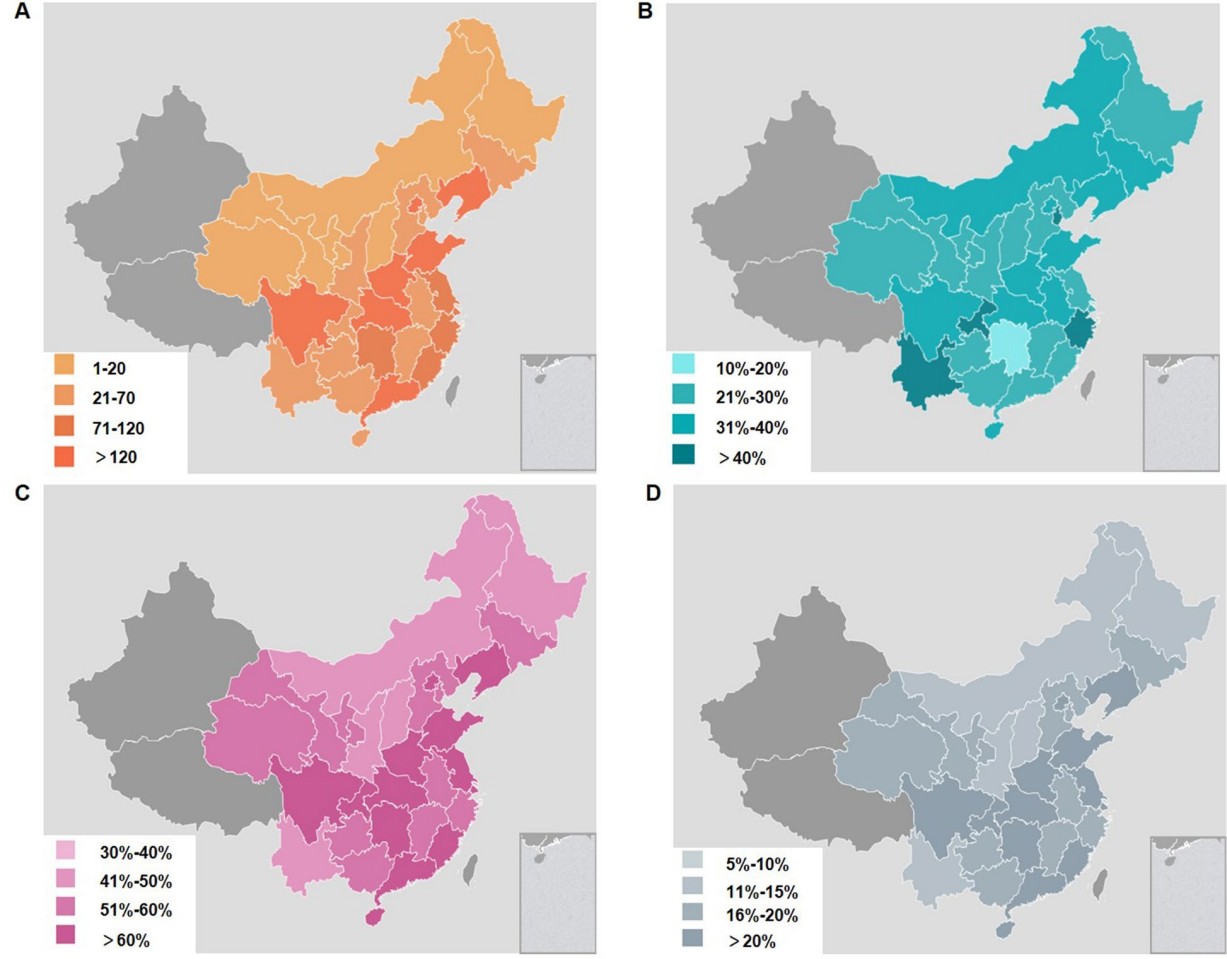
Number of questionnaires submitted and proportion of procedural analgesic interventions centers distributed in each region. (**A**) Number of questionnaires submitted by each region. (**B**) Proportion of procedural analgesic interventions centers by each region. (**C**) Population’s surveying ratio of Grade III hospitals. (**D**) Population’s surveying ratio of Grade II hospitals

A total of 2198 (44.0%) valid questionnaires from 29 municipalities, provinces and autonomous regions were included in this study as we reported previously [9], and the proportion of questionnaires submitted by each region is shown in Fig. 1A. The hospitals responding to our survey were mainly Grade III hospitals (1418/2198, 64.51%), while 780 (780/2198, 35.49%) were Grade II class hospitals. (Table 1). There were 730 (33.2%) hospitals that established procedural analgesic interventions centers, and details on the procedural analgesic interventions centers’ distribution in mainland China are shown in Fig. 1B. In addition, Fig. 1C and D showed the population’s surveying ratio of Grade III and Grade II hospitals, respectively.

**Table 1:** Characteristics of the surveyed hospitals

	Number of Hospitals, *N* (%)
Hospital grade	
Grade III	1418 (64.5%)
Grade II	780 (35.5%)
Hospital type	
General hospital	1755 (79.9%)
Specialized hospital	141 (6.4%)
Maternal and child care service center	158 (7.2%)
Tumor hospital	46 (2.1%)
Chest hospital	14 (0.6%)
Stomatological hospital	12 (0.6%)
Children’s hospital	30 (1.4%)
Others	42 (1.9%)

### Current status of procedural analgesic interventions in Chinese hospitals

**Fig. 2:**
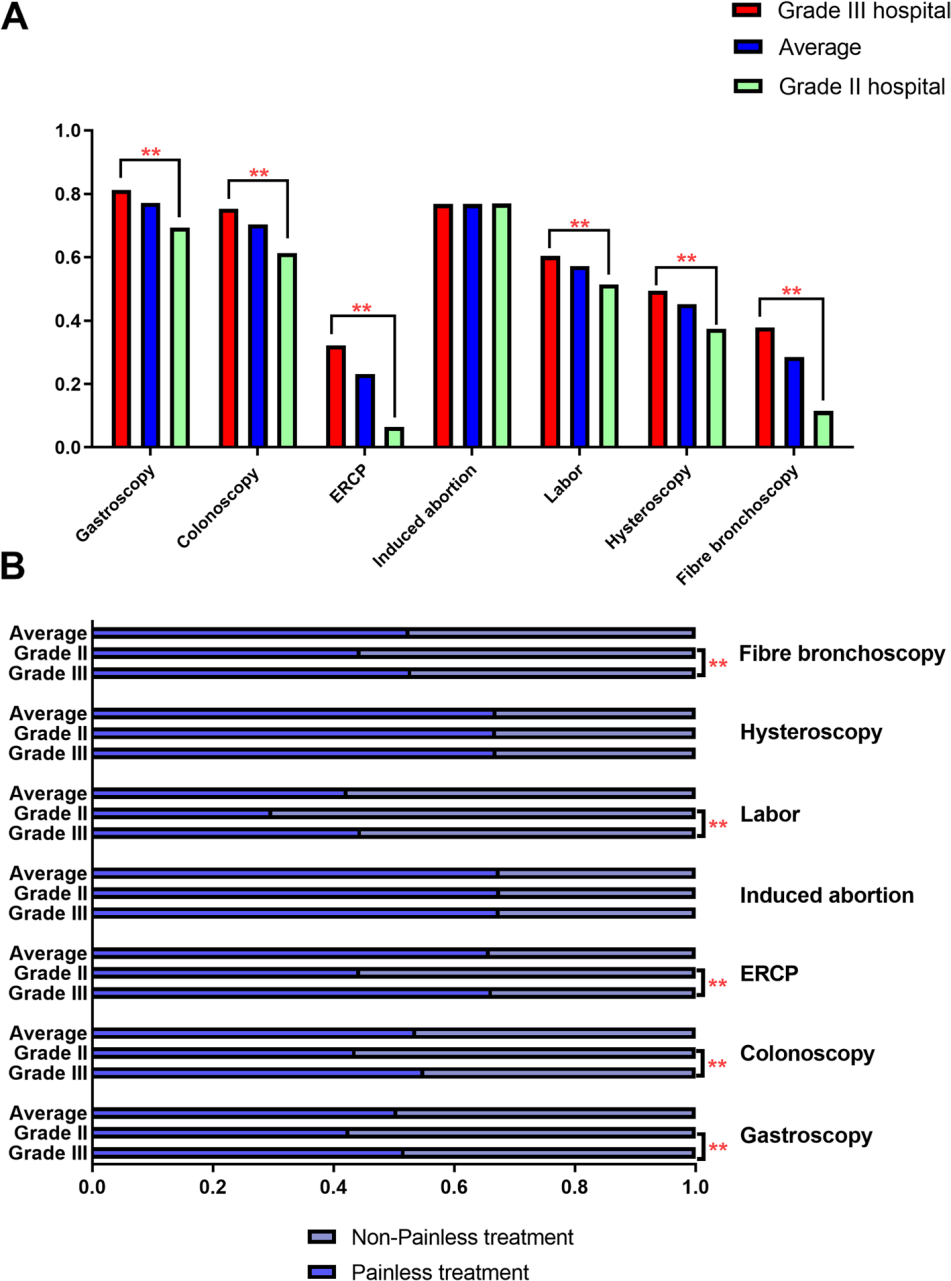
Proportion of different kinds of procedural analgesic interventions in surveyed hospitals. (**A**) Percentage of surveyed hospitals providing procedural analgesic interventions. (**B**) Percentage of procedural analgesic interventions among the surveyed hospitals. ** *p* < 0.01 in comparisons with the Grade III hospital group

In total, 2101 (95.6%) hospitals provided at least one of the surveyed procedural analgesic interventions services, including gastroscopy (77.1%), colonoscopy (70.3%), ERCP (23.1%), induced abortion (76.8%), labor (57.2%), hysteroscopy (45.1%) and fiber bronchoscopy (28.5%). Compared with Grade II hospitals, Grade III hospitals had a higher proportion of procedural analgesic interventions services for gastroscopy (81.3% vs. 69.4%, *p* < 0.001), colonoscopy (75.3% vs. 61.3%, *p* < 0.001), ERCP (32.5% vs. 6.5%, *p* < 0.001), labor (60.4% vs. 51.4%, *p *< 0.001), hysteroscopy (49.4% vs. 37.4%, *p* < 0.001), and fiber bronchoscopy (37.9% vs. 11.5%, *p* < 0.001) (Fig. 2A). However, no difference was observed in induced abortion among the different grades of hospitals (76.8% vs. 76.9%, *p* = 0.958). The overall sedation rate was 50.6% for gastroscopy, 53.7% for colonoscopy, 65.9% for ERCP, 67.5% for induced abortion, 42.3% for labor, 67.0% for hysteroscopy and 52.6% for fiber bronchoscopy. We found that procedural analgesic intervention of gastroscopy (51.8% vs. 42.6%, *p* < 0.001), colonoscopy (55.0% vs. 43.7%, *p* < 0.001), ERCP (66.3% vs. 44.4%, *p* < 0.001), labor (44.6% vs. 29.8%, *p* < 0.001) and fiber bronchoscopy (52.9% vs. 44.5%, *p* < 0.001) accounted for a larger proportion in Grade III hospitals relative to Grade II hospitals (Fig. 2B).

### Anesthesiologists for procedural analgesic interventions in Chinese hospitals

**Fig. 3:**
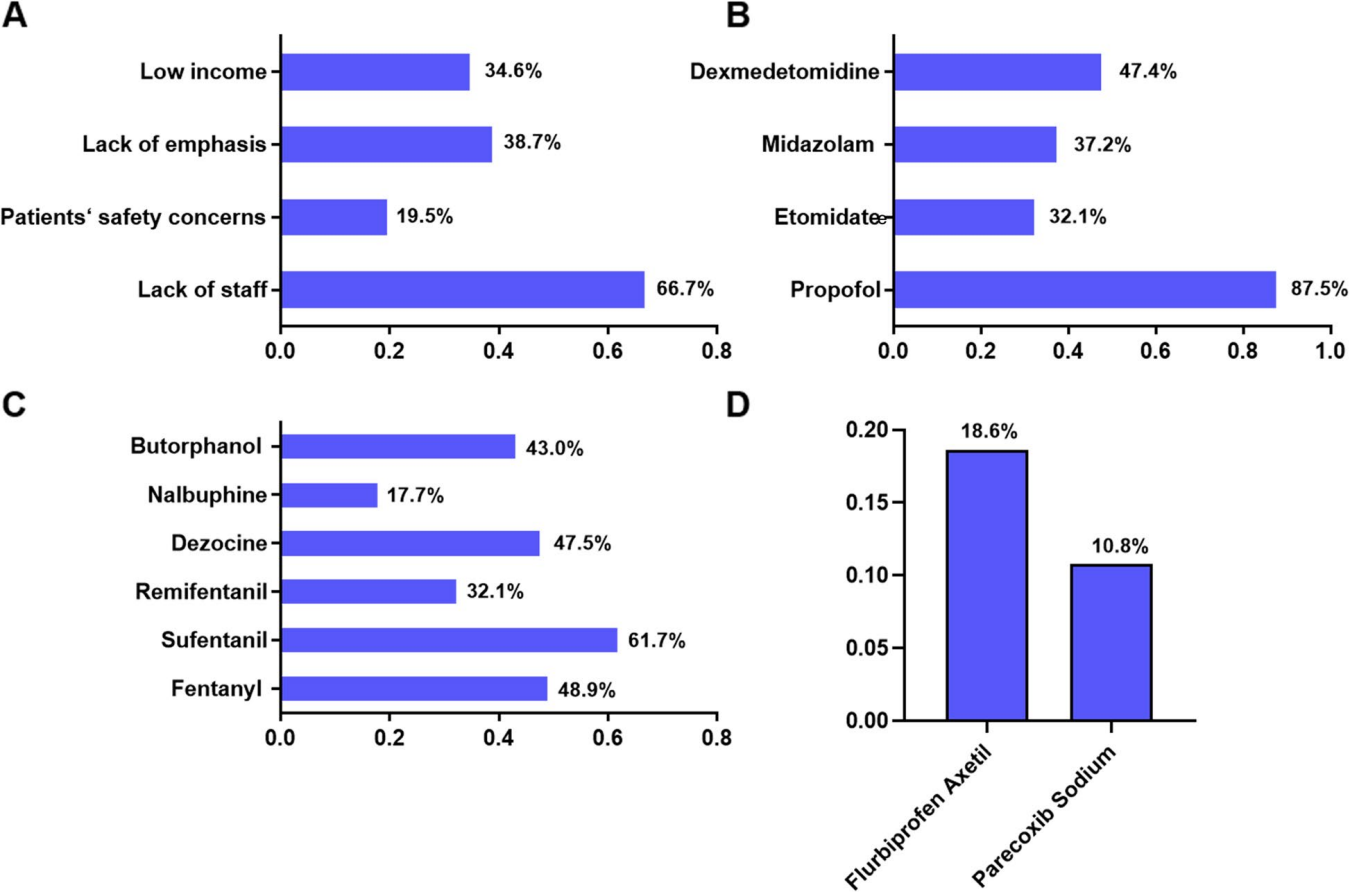
Difficulties and sedative and analgesic use during procedural analgesic interventions among the surveyed hospitals. (**A**) Difficulties during procedural analgesic interventions. (**B**) Sedative use during procedural analgesic interventions. (**C**) Opioids use during procedural analgesic interventions. (**D**) NSAIDs use during procedural analgesic interventions

A Consensus Statement of 21 European National Societies of Anesthesia has suggested that non-anesthesiologists should not be allowed to administer propofol for procedural sedation [14]. In mainland China, only anesthesiologists are allowed to perform sedation and analgesia for procedural analgesic interventions according to the policy and Clinical guidelines (Chinese Guideline for Painless Digestive Endoscopy and Expert consensus on anesthesia management for common digestive endoscopic surgery) [15]. On average (median [IQR]), each anesthesiologist performed 5.7 [2.3—11.4] cases per day. This value was 7.3 [3.2—13.6] in Grade III hospitals and 3.4 [1.8—6.8] in Grade II hospitals (z = -7.065, *p* < 0.001) (calculated over 22 working days per month). These results revealed that anesthesiologists in Grade III hospitals experienced greater work pressure associated with procedural analgesic interventions. In addition, we found that a lack of staff (66.7%), lack of emphasis (38.7%), low income (34.6%) and patient safety concerns (19.5%) were the main barriers for procedural analgesic interventions (Fig. 3A).

### Sedation drugs and analgesics for procedural analgesic interventions in Chinese hospitals

As shown in Fig. 3B, the most frequently used sedation drugs were propofol (87.5%), dexmedetomidine (47.4%), midazolam (37.2%) and etomidate (32.1%). As shown in Fig. 3C and D, the favored analgesics were sufentanil (61.7%), fentanyl (48.9%), and dezocine (47.5%), followed by butorphanol (43.0%), remifentanil (32.1%), flurbiprofen axetil (18.6%), nalbuphine (17.7%) and parecoxib sodium (10.8%).

## Discussion

In this national survey, which included a total of 2198 hospitals across mainland China, we revealed the current status of procedural analgesic interventions in China. Our results suggested that the ratio of procedural analgesic interventions was relatively low. Moreover, compared with Grade II hospitals, the Grade III hospitals had a higher proportion of procedural analgesic interventions services except during induced abortion.

Based on our results, three-quarters of the surveyed hospitals provide procedural analgesic interventions of gastroscopy and colonoscopy, although the overall sedation rates of gastroscopy and colonoscopy were relatively low. A possible explanation for this is that outpatient procedures were not covered by medical insurance and patients may choose examinations without sedation for economic concerns. In addition, Yang et al. suggested that concern about sedation was associated with anxiety during colonoscopy which may also contribute to a low sedation rate [16]. More importantly, these situations may occur for other types of examinations or treatments. Interventions designed to increase the amount of education in various formats received by patients before examination represent promising strategies to reduce anxiety and increase the sedation ratio.

It is reasonable for Grade III hospitals to take more responsibilities because the educational background required for these hospitals is greater than that for Grade II hospitals according to a national survey in China [1]. However, the volumes of Grade III hospitals are more than three times those of Grade II hospitals. Anesthesiologists in Grade III hospitals experienced greater work pressure regarding procedural analgesic interventions services, suggesting an uneven distribution and utilization of medical resources. This finding is in accordance with observations by Zhou et al. [15].

### Procedural analgesic intervention of gastrointestinal endoscopy

A survey of 2758 Chinese hospitals in 2016 showed that sedation was used with gastroscopy (47.9%) and colonoscopy (49.3%), which suggests that the sedation rate for gastrointestinal endoscopy is much lower in China than in the US and Europe [15]. Our results indicate that the sedation rate for gastrointestinal endoscopy (50.6% in gastroscopy and 53.7% in colonoscopy) has increased slowly over the past three years, which may be related to the rapid increase in volumes [1].

For ERCP, Hu et al. reported that 24.4% of ERCP procedures in 2013 were performed with patients under conscious sedation, while our results showed that 65.9% of these procedures in 2019 were performed with sedation/analgesia [17]. Similarly, most ERCP procedures with or without sedation were performed in Grade III hospitals. Since the General Office of the State Council promulgated the construction of a hierarchical medical system (aiming to improve services at county- and township-level health centers, especially in less-developed areas), this uneven use of health care mentioned above will gradually improve [18].

### Procedural analgesic interventions of gynaecology and obstetrics

Neuraxial analgesia is considered the most effective method for reducing pain during labor and decreasing the risk of postpartum depression [19, 20]. However, the historical estimated overall prevalence of neuraxial analgesia use in China was 10% [21], while this ratio in France and the United States was 88% and 73%, respectively [4, 22]. Meanwhile, the rate of cesarean delivery in China was among the highest worldwide in 2007 (46%) and 2014 (35%) [23–25]. The high rate of caesarean section may be explained by medical, social, cultural and individual factors, and can also be influenced by family members and health professionals [26]. And the two-child policy may result an increased rate cause 90% of women with previous caesarean section eventually gave birth by caesarean section [27]. Since the National Health Commission issued two policies in 2018 to promote labor neuraxial analgesia in China, the estimated national labor neuraxial analgesia rates increased from 8.4% in 2012 to 16.7% in 2019 [8, 28, 29]. Our results showed that more than half of the surveyed hospitals provide analgesia during labor, with a ratio of 42.3% of parturients receiving analgesia, which is much higher than the value of 16.7% reported in 2019. This inconsistency may be due to the sample size and different proportions of Grade III hospitals. According to the National Health Service and Quality and Safety Report in 2019, 31.7% of parturients received neuraxial analgesia, which is similar to our results [30]. The low rate of epidural analgesia for labor is mainly because of lacking anesthesiologists. The number of anesthesiologists per 100,000 of the population in China was 6.89/100000 in 2019 and still far from high-income countries (17.96/100000) [1, 31].

A meta-analysis suggested that pain during uterine interventions performed when the patient was awake was unacceptable [32]. Although some gynecologists believe that too much emphasis is placed on the issue of pain surrounding outpatient hysteroscopy because most patients do not experience considerable pain, the minimal discomfort experienced by the patient is considered a trade-off for the convenience and interaction associated with outpatient hysteroscopy [33]. The utilization of local anesthesia alone for hysteroscopy is inadequate and often leads to additional sedation rates, which suggests that analgesics are routinely used under general anesthesia as a supplement to local anesthetics [34–36]. Our results showed that among hospitals that provide hysteroscopy with sedation and analgesics, the sedation/analgesia rate is the highest. For induced abortion, the condition is similar to that for hysteroscopy.

### Procedural analgesic interventions of fibre bronchoscopy

Fiber bronchoscopy is an important method for the clinical diagnosis and treatment of respiratory diseases that present high stimulus intensity, hypoxemia, and strong patient discomfort. Sedation/analgesia can improve the tolerance of patients undergoing this procedure and provide better examination conditions. It has been suggested that a small percentage of hospitals perform fiber bronchoscopy, and most patients receive general anesthesia [37]. In Switzerland, the sedation rate during fiber bronchoscopy was 95%, although in our results for China, this rate was only 52.6% on average [38]. However, considering the number of hospitals that do not offer procedural analgesic interventions of bronchoscopy in China, this ratio drops dramatically. The development of bronchoscopy in China is uneven by hospital level and region [39].

This study has several limitations. Firstly, our questionnaire survey obtained information from anesthesiologists and failed to capture patients’ responses; hence, the responses may lack complete feedback. Secondly, our national survey only included the chief anesthesiologist or a member of the Pain Group of the Chinese Society of Anesthesiology in each identified hospital. This method was warrant of a good response rate and the chief anesthesiologist would have better insight into their frame of work due to that the chief regularly collected quality control data on procedural analgesic interventions [40, 41]. In some section, such as barriers in practice, may be reported more readily by an individual than by a chief. However, the chief anesthesiologists may treat the barriers from a higher position (on the side of the department even the Chinese anesthesiology).

## Conclusion

Chinese anesthesiologists have made great efforts toward procedural analgesic interventions, as evidenced by the increasing rate compared to past surveys. However, a large gap remains between China and developed countries. It may be meaningful to further explore the rate of procedural analgesic interventions and its influencing factors in various hospitals to increase the proportion and benefit more patients. The uneven use of health care at the hospital and regional levels and the lack of anesthesiologists are the main barriers to optimal procedural analgesic interventions.

## Supplementary Information

**Additional file 1.**

**Additional file 2.**

## Data Availability

The data used to support the findings of this study are included within the article.
