# Supplementary material for: Procedural analgesic interventions in China: a national survey of 2198 hospitals
Source: BMC Anesthesiol. 2022 Aug 6;22:250. doi: 10.1186/s12871-022-01783-6 (PMC9356406; doi:10.1186/s12871-022-01783-6)
Supplement: Supplementary file 1 — Additional file 1. [file 12871_2022_1783_MOESM1_ESM.docx]

**无痛诊疗调研问卷**

第1题 医院名称：

第2题 医院等级

[单选题]

1. 三级
2. 二级

第3题 医院类型

[单选题]

A. 综合性医院

B. 专科医院

C. 肿瘤医院

D. 妇幼保健医院

E. 胸科医院

F. 口腔医院

G. 儿童医院

H. 其他

第4题 医院编制床位______张

第5题 医院年门诊量：________

第6题 医院年手术量：________

第7题 麻醉科医生（人）：________

第8题 是否开展无痛诊疗：

[单选题]

A. 是

B. 否

第9题 是否有独立的无痛诊疗中心：

[单选题]

A. 是

B. 否

第10题 是否有独立的无痛诊疗小组：

[单选题]

A. 是

B. 否

第11题 无痛中心床位数（个）________

第12题 无痛诊疗数（例/月）________

第13题 无痛诊疗医务人员数（人）________

第14题 无痛诊疗是否专人负责？

[单选题]

1. 是
2. 否

第15题 多长时间轮换？________

第16题 无痛诊疗操作包括哪些？

[多选题]

1. 不开展
2. 无痛胃镜
3. 无痛肠镜
4. 无痛ERCP
5. 无痛人流
6. 无痛分娩
7. 无痛宫腔镜
8. 无痛纤支镜

第17题 无痛胃镜诊疗______例数/月，普通______例数/月

第18题 无痛肠镜诊疗______例数/月，普通______例数/月

第19题 无痛ERCP______例数/月，普通______例数/月

第20题 无痛人流______例数/月，普通______例数/月

第21题 无痛分娩______例数/月，普通______例数/月

第22题 无痛宫腔镜______例数/月，普通______例数/月

第23题 无痛纤支镜______例数/月，普通______例数/月

第24题 开展无痛诊疗最大的障碍是什么：

[多选题]

1. 收费过低
2. 麻醉医生短缺
3. 医院不重视
4. 患者认为不安全

第25题 麻醉镇静药物：

[多选题]

A. 丙泊酚

B. 依托咪酯

C. 咪达唑仑

D. 右美托咪定

第26题 镇痛药物：

[多选题]

1. 帕瑞昔布钠
2. 芬太尼
3. 舒芬太尼
4. 瑞芬太尼
5. 地佐辛
6. 纳布啡
7. 布托啡诺
8. 氟比洛芬酯
