# Supplementary material for: Procedural analgesic interventions in China: a national survey of 2198 hospitals
Source: BMC Anesthesiol. 2022 Aug 6;22:250. doi: 10.1186/s12871-022-01783-6 (PMC9356406; doi:10.1186/s12871-022-01783-6)
Supplement: Supplementary file 2 — Additional file 2. [file 12871_2022_1783_MOESM2_ESM.docx]

**Survey Questionnaire**

1. Institution：
2. The grades of hospital

[Single choice]

A. Grade III

B. Grade II

3. The types of Hospital

[Single choice]

A. General hospital

B. Specialized hospital

C. Maternal and child care service center

D. Tumor hospital

E. Chest hospital

F. Stomatological hospital

G. Children’s hospital

H. Others

4. Number of hospital beds________

5. Number of outpatients per year：________

6. Number of surgeries per year：________

7.Number of anesthesiologists：________

8. Whether provide service of procedural analgesic interventions：

[Single choice]

A. Yes

B. No

9. Whether procedural analgesic interventions centers were established

[Single choice]

A. Yes

B. No

10. Whether there is an independent anesthesiologists group for procedural analgesic interventions：

[Single choice]

A. Yes

B. No

11. Number of hospital beds in procedural analgesic interventions centers ________

12. Number of procedural analgesic interventions cases per month ________

13. Number of medical staff in procedural analgesic interventions centers ________

14. Whether permanent staffs responsible for procedural analgesic interventions [Single choice]

A. Yes

B. No

15. How long is the rotation? ________

16. Procedural analgesic interventions include:

[Multiple choice]

1. None
2. Procedural analgesic intervention of gastroscopy
3. Procedural analgesic intervention of colonoscopy
4. Procedural analgesic intervention of ERCP
5. Procedural analgesic intervention of induced abortion
6. Procedural analgesic intervention of labor
7. Procedural analgesic intervention of hysteroscopy
8. Procedural analgesic intervention of fibre bronchoscopy

17.

Procedural analgesic intervention of gastroscopy ______cases per month.

Non-procedural analgesic intervention of gastroscopy ______cases per month.

18.

Procedural analgesic intervention of colonoscopy ______cases per month.

Non- procedural analgesic intervention of colonoscopy ______cases per month.

19.

Procedural analgesic intervention of ERCP ______cases per month.

Non- procedural analgesic intervention of ERCP ______cases per month.

20.

Procedural analgesic intervention of induced abortion ______cases per month.

Non- procedural analgesic intervention of induced abortion ______cases per month.

21.

Procedural analgesic intervention of labor ______cases per month.

Non- procedural analgesic intervention of labor ______cases per month.

22.

Procedural analgesic intervention of hysteroscopy ______cases per month.

Non- procedural analgesic intervention of hysteroscopy ______cases per month.

23.

Procedural analgesic intervention of fibre bronchoscopy ______cases per month.

Non- procedural analgesic intervention of fibre bronchoscopy ______cases per month.

24. Barriers for procedural analgesic interventions [Multiple choice]

1. Low income
2. Lack of staff
3. Lack of emphasis
4. Patients’ safety concerns

25. Sedation drugs for procedural analgesic interventions：

[Multiple choice]

A. Propofol

B. Etomidate

C. Midazolam

D. Dexmedetomidine

26. Analgesics for procedural analgesic interventions：

[Multiple choice]

1. Parecoxib Sodium
2. Fentanyl
3. Sufentanil
4. Remifentanil
5. Dezocine
6. Nalbuphine
7. Butorphanol
8. Flurbiprofen Axetil
